# (3-Benzoyl-1,7,7-trimethyl­bicyclo­[2.2.1]heptan-2-olato-κ^2^ *O*,*O*)bis­[2-(2-pyrid­yl)phenyl-κ^2^ *C* ^1^,*N*]iridium(III)

**DOI:** 10.1107/S1600536811039262

**Published:** 2011-10-12

**Authors:** Kaijun Luo, Juan Jia, Yanfang Chen, Daibing Luo

**Affiliations:** a College of Chemistry and Materials Science, Sichuan Normal University, Chengdu, Sichuan 610068, People’s Republic of China; b Analytical and Testing Center, Sichuan University, Chengdu, Sichuan 610065, People’s Republic of China

## Abstract

The title compound, [Ir(C_11_H_8_N)_2_(C_17_H_19_O_2_)], has an octa­hedral coordination geometry around the Ir^III^ atom, retaining the *cis*-*C*,*C*,*trans*–*N*,*N* chelate disposition of the two 2-phenyl­pyridine ligands. The chelate rings are nearly mutually perpendicular [the inter­planar angles range from 85.48 (17) to 89.17 (19)°]. The two 2-(2-pyrid­yl)phenyl ligands are approximately planar, with the plane of the phenyl ring being inclined to that of the pyridine ring by 2.3 (3) and 5.1 (3)° in the two ligands. The inter­planar angle between the phenyl ring in 3-benzoyl-camphor and the IrO_2_C_3_ chelate ring is 35.5 (2)°.

## Related literature

For general background and for related structures, see: Ulbricht *et al.* (2009 ▶); Lamansky *et al.* (2001*a* ▶); Jones *et al.* (2010 ▶). For the synthesis of 3-benzoyl-camphor and the title complex, see: Tamiaki *et al.* (2003 ▶); Lamansky *et al.* (2001*b* ▶); Luo *et al.* (2011 ▶).
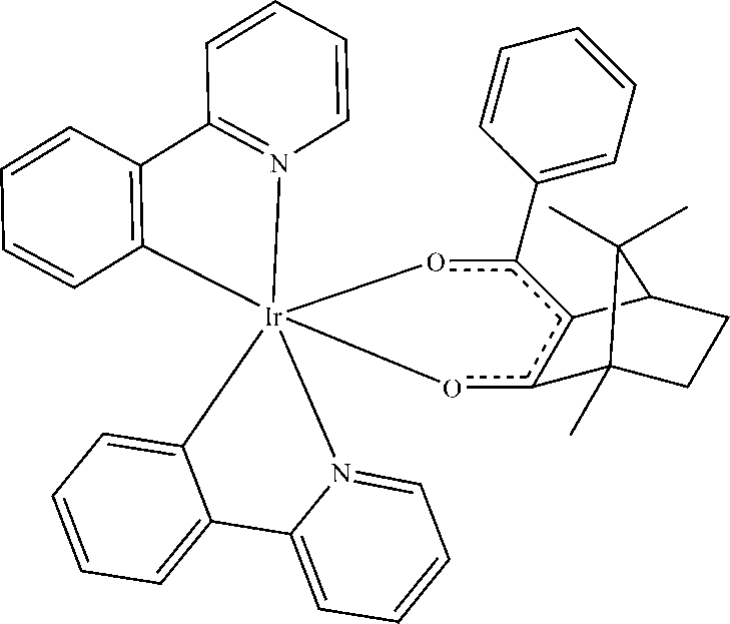


## Experimental

### 

#### Crystal data

[Ir(C_11_H_8_N)_2_(C_17_H_19_O_2_)]
*M* *_r_* = 755.89
Monoclinic, 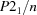

*a* = 10.4569 (6) Å
*b* = 15.0979 (9) Å
*c* = 19.9110 (11) Å
β = 101.755 (6)°
*V* = 3077.6 (3) Å^3^
*Z* = 4
Mo *K*α radiation
μ = 4.38 mm^−1^
*T* = 296 K
0.36 × 0.32 × 0.23 mm

#### Data collection

Oxford Diffraction Xcalibur Eos diffractometer
Absorption correction: multi-scan (*CrysAlis PRO*; Oxford Diffraction, 2010 ▶) *T*_min_ = 0.302, *T*_max_ = 0.433
12765 measured reflections
6233 independent reflections
4631 reflections with *I* > 2σ(*I*)
*R*_int_ = 0.032

#### Refinement

*R*[*F* ^2^ > 2σ(*F* ^2^)] = 0.038
*wR*(*F* ^2^) = 0.076
*S* = 0.97
6233 reflections
400 parameters
H-atom parameters constrained
Δρ_max_ = 1.21 e Å^−3^
Δρ_min_ = −1.31 e Å^−3^

### 

Data collection: *CrysAlis PRO* (Oxford Diffraction, 2010 ▶); cell refinement: *CrysAlis PRO*; data reduction: *CrysAlis PRO*; program(s) used to solve structure: *SHELXS97* (Sheldrick, 2008 ▶); program(s) used to refine structure: *SHELXL97* (Sheldrick, 2008 ▶); molecular graphics: *OLEX2* (Dolomanov *et al.*, 2009 ▶); software used to prepare material for publication: *OLEX2*.

## Supplementary Material

Crystal structure: contains datablock(s) global, I. DOI: 10.1107/S1600536811039262/su2315sup1.cif

Structure factors: contains datablock(s) I. DOI: 10.1107/S1600536811039262/su2315Isup2.hkl

Additional supplementary materials:  crystallographic information; 3D view; checkCIF report

## Figures and Tables

**Fig. 1.**
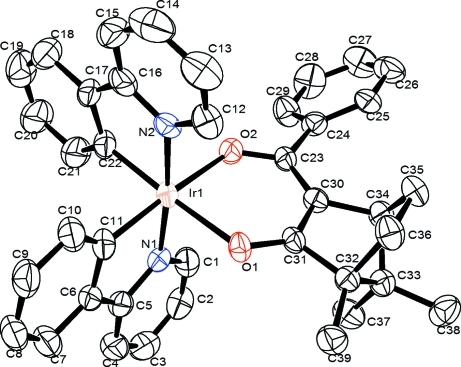
The molecular structure of the title complex, with atom labels and 50% probability displacement ellipsoids. Hydrogen atoms have been omitted for clarity.

## supplementary crystallographic information

### Comment

The phosphorescent cyclometalated Iridium(III) complexes have recently received considerable attention in the fabrication of phosphorescent organic light emitting diodes [OLED's] for their high quantum efficiencies, and relatively short phosphorescent lifetimes. We have synthesized a novel cyclometalated Iridium(III) complex with 3-benzoyl-camphor as ancillary ligand, and we report herein on its crystal structure.

The molecular structure of the title complex is shown in Fig. 1. The coordination at the iridium atom is octahedral. In comparison with a similar complex, bis(2-pyridylphenyl)(acetylacetonate)iridium(III) [Ir(ppy)_2_(acac)] (Lamansky *et al.*, 2001*a*), the title complex displays longer Ir—N [2.032 (4) and 2.033 (4) Å] and Ir—O [2.153 (3) and 2.171 (3) Å] bond distances than those in Ir(ppy)_2_(acac) [Ir—N = 1.996 (5) and 2.001 (5) Å, and Ir—O = 2.003 (9) and 2.003 (9) Å]. In contrast the Ir—C bonds in the title complex [1.996 (5) and 2.001 (5) Å] are similar to those in Ir(ppy)_2_(acac) [2.003 (9) and 2.003 (9) Å].

The interplanar angles between the chelate rings, for the IrO_2_C_3_ ring A (Ir1,O1,C31,C30,C23,O2) to the IrNC_3_ rings B (Ir1,N1,C5,C6,C11) and C (Ir1,N2,C16,C17,C22) are 85.48 (17) and 86.79 (16) °, respectively, while the IrNC_3_ rings, B and C, are inclined at an angle of 89.17 (19) °. The dihedral angles between the pyridyl and phenyl rings of the 2-phenylpyridine ligands, involving atoms N1 and N2, are 2.3 (3) and 5.1 (3)°, respectively. The interplanar angle between the phenyl ring (C24-C29) in 3–benzoyl–camphor and the IrO_2_C_3_ chelate ring A is 35.5 (2) °.

### Experimental

The 3–benzoyl–camphor was prepared according to the method of (Tamiaki *et al.*, 2003). The Ir(III) µ–dichloro–bridged dimer, [IrCl(ppy)_2_]_2_, was prepared according to the method of (Lamansky *et al.*, 2001*b*). To a solution of 3–benzoyl–camphor (5.0 mmol, 1.27 g) in 2–ethoxyethanol (30 ml), were added [IrCl(ppy)_2_]_2_ (2 mmol, 2.14 g) and anhydrous Cs_2_CO_3_ (2 mmol, 0.652 g). The mixture was stirred under an inert atmosphere at 373 K for 20 h. After cooling to room temperature, the resulting solution was filtered to remove residual Cs_2_CO_3_ and washed with water, and then concentrated under reduced pressure. The residue was purified by chromatography on silica gel [eluent: dichloromethane and petroleum ether, v/v = 2:3] Yellow crystals, suitable for X-ray diffraction analysis, were obtained by slow diffusion of methanol into a dichloromethane solution of the title complex.

### Refinement

H atoms were placed at calculated positions and treated as riding atoms: C—H = 0.93, 0.98, 0.97 and 0.96 Å for CH(aromatic), CH(methine), CH_2_ and CH_3_ H-atoms, respectively, with *U*_iso_(H) = k × U_eq_(C), where k = 1.5 for CH_3_ H atoms, and k = 1.2 for all other H atoms.

### Crystal data

**Table:**

[Ir(C_11_H_8_N)_2_(C_17_H_19_O_2_)]	*F*(000) = 1504
*M**_r_* = 755.89	*D*_x_ = 1.631 Mg m^−^^3^
Monoclinic, *P*2_1_/*n*	Mo *K*α radiation, λ = 0.71073 Å
Hall symbol: -P 2yn	Cell parameters from 6379 reflections
*a* = 10.4569 (6) Å	θ = 2.9–28.5°
*b* = 15.0979 (9) Å	µ = 4.38 mm^−^^1^
*c* = 19.9110 (11) Å	*T* = 296 K
β = 101.755 (6)°	Block, yellow
*V* = 3077.6 (3) Å^3^	0.36 × 0.32 × 0.23 mm
*Z* = 4	

### Data collection

**Table:**

Oxford Diffraction Xcalibur Eos diffractometer	6233 independent reflections
Radiation source: Enhance (Mo) X-ray Source	4631 reflections with *I* > 2σ(*I*)
graphite	*R*_int_ = 0.032
Detector resolution: 16.0874 pixels mm^-1^	θ_max_ = 26.4°, θ_min_ = 2.9°
ω scans	*h* = −13→12
Absorption correction: multi-scan (*CrysAlis PRO*; Oxford Diffraction, 2010)	*k* = −16→18
*T*_min_ = 0.302, *T*_max_ = 0.433	*l* = −22→24
12765 measured reflections	

### Refinement

**Table:**

Refinement on *F*^2^	0 restraints
Least-squares matrix: full	H-atom parameters constrained
*R*[*F*^2^ > 2σ(*F*^2^)] = 0.038	*w* = 1/[σ^2^(*F*_o_^2^) + (0.0396*P*)^2^] where *P* = (*F*_o_^2^ + 2*F*_c_^2^)/3
*wR*(*F*^2^) = 0.076	(Δ/σ)_max_ = 0.002
*S* = 0.97	Δρ_max_ = 1.21 e Å^−^^3^
6233 reflections	Δρ_min_ = −1.31 e Å^−^^3^
400 parameters	

### Special details

**Table:**

Experimental. (CrysAlis PRO; Oxford Diffraction, 2010). Empirical absorption correction using spherical harmonics, implemented in SCALE3 ABSPACK scaling algorithm.
Geometry. All e.s.d.'s (except the e.s.d. in the dihedral angle between two l.s. planes) are estimated using the full covariance matrix. The cell e.s.d.'s are taken into account individually in the estimation of e.s.d.'s in distances, angles and torsion angles; correlations between e.s.d.'s in cell parameters are only used when they are defined by crystal symmetry. An approximate (isotropic) treatment of cell e.s.d.'s is used for estimating e.s.d.'s involving l.s. planes.
Refinement. Refinement of *F*^2^ against ALL reflections. The weighted *R*-factor *wR* and goodness of fit *S* are based on *F*^2^, conventional *R*-factors *R* are based on *F*, with *F* set to zero for negative *F*^2^. The threshold expression of *F*^2^ > σ(*F*^2^) is used only for calculating *R*-factors(gt) *etc*. and is not relevant to the choice of reflections for refinement. *R*-factors based on *F*^2^ are statistically about twice as large as those based on *F*, and *R*- factors based on ALL data will be even larger.

### Fractional atomic coordinates and isotropic or equivalent isotropic displacement parameters (Å^2^)

**Table:**

	*x*	*y*	*z*	*U*_iso_*/*U*_eq_	
Ir1	0.011297 (17)	0.240633 (12)	0.384356 (9)	0.03589 (7)	
O1	0.1844 (3)	0.1580 (2)	0.39631 (15)	0.0355 (7)	
O2	0.0088 (3)	0.2488 (2)	0.27613 (16)	0.0410 (8)	
N1	−0.0946 (4)	0.1267 (3)	0.3756 (2)	0.0411 (10)	
N2	0.1116 (4)	0.3565 (3)	0.40134 (18)	0.0354 (9)	
C1	−0.1432 (5)	0.0837 (4)	0.3165 (3)	0.0483 (13)	
H1	−0.1273	0.1073	0.2758	0.058*	
C2	−0.2153 (5)	0.0065 (4)	0.3132 (3)	0.0551 (14)	
H2	−0.2492	−0.0206	0.2715	0.066*	
C3	−0.2354 (5)	−0.0293 (4)	0.3753 (3)	0.0571 (16)	
H3	−0.2805	−0.0823	0.3758	0.069*	
C4	−0.1876 (5)	0.0151 (4)	0.4351 (3)	0.0536 (14)	
H4	−0.2033	−0.0078	0.4760	0.064*	
C5	−0.1169 (4)	0.0924 (3)	0.4370 (3)	0.0431 (12)	
C6	−0.0578 (4)	0.1448 (3)	0.4969 (2)	0.0397 (12)	
C7	−0.0643 (5)	0.1208 (4)	0.5638 (3)	0.0518 (14)	
H7	−0.1059	0.0685	0.5718	0.062*	
C8	−0.0086 (5)	0.1750 (4)	0.6182 (3)	0.0574 (15)	
H8	−0.0134	0.1596	0.6629	0.069*	
C9	0.0532 (6)	0.2509 (4)	0.6057 (3)	0.0551 (15)	
H9	0.0917	0.2871	0.6420	0.066*	
C10	0.0588 (5)	0.2743 (4)	0.5394 (3)	0.0502 (13)	
H10	0.1004	0.3268	0.5321	0.060*	
C11	0.0050 (5)	0.2227 (3)	0.4832 (2)	0.0423 (12)	
C12	0.2430 (5)	0.3628 (4)	0.4152 (3)	0.0501 (14)	
H12	0.2923	0.3123	0.4121	0.060*	
C13	0.3068 (6)	0.4420 (5)	0.4339 (3)	0.0630 (16)	
H13	0.3975	0.4450	0.4425	0.076*	
C14	0.2339 (7)	0.5162 (4)	0.4394 (3)	0.0696 (18)	
H14	0.2742	0.5700	0.4529	0.083*	
C15	0.0998 (6)	0.5094 (4)	0.4245 (3)	0.0545 (14)	
H15	0.0492	0.5593	0.4275	0.065*	
C16	0.0404 (5)	0.4308 (4)	0.4054 (2)	0.0467 (13)	
C17	−0.1026 (5)	0.4141 (3)	0.3877 (2)	0.0450 (13)	
C18	−0.1962 (6)	0.4818 (4)	0.3824 (3)	0.0619 (16)	
H18	−0.1709	0.5406	0.3900	0.074*	
C19	−0.3295 (6)	0.4585 (5)	0.3653 (3)	0.0695 (18)	
H19	−0.3933	0.5021	0.3623	0.083*	
C20	−0.3647 (6)	0.3725 (4)	0.3531 (3)	0.0688 (18)	
H20	−0.4527	0.3576	0.3420	0.083*	
C21	−0.2731 (5)	0.3079 (4)	0.3569 (3)	0.0592 (16)	
H21	−0.3009	0.2499	0.3475	0.071*	
C22	−0.1386 (5)	0.3247 (3)	0.3746 (2)	0.0399 (12)	
C23	0.0909 (4)	0.2123 (3)	0.2447 (2)	0.0330 (10)	
C24	0.0621 (4)	0.2298 (3)	0.1683 (2)	0.0365 (11)	
C25	0.1607 (5)	0.2425 (3)	0.1319 (2)	0.0413 (11)	
H25	0.2478	0.2387	0.1541	0.050*	
C26	0.1288 (6)	0.2608 (3)	0.0621 (3)	0.0556 (14)	
H26	0.1952	0.2686	0.0379	0.067*	
C27	0.0046 (6)	0.2674 (4)	0.0291 (3)	0.0634 (17)	
H27	−0.0148	0.2773	−0.0180	0.076*	
C28	−0.0949 (6)	0.2597 (4)	0.0646 (3)	0.0647 (17)	
H28	−0.1812	0.2677	0.0421	0.078*	
C29	−0.0658 (5)	0.2400 (4)	0.1336 (3)	0.0523 (14)	
H29	−0.1333	0.2334	0.1572	0.063*	
C30	0.1920 (4)	0.1572 (3)	0.2748 (2)	0.0337 (10)	
C31	0.2301 (4)	0.1346 (3)	0.3445 (2)	0.0311 (10)	
C32	0.3490 (4)	0.0745 (3)	0.3523 (2)	0.0349 (11)	
C33	0.3151 (4)	0.0220 (3)	0.2842 (2)	0.0377 (11)	
C34	0.2894 (4)	0.1080 (3)	0.2407 (2)	0.0377 (11)	
H34	0.2600	0.0984	0.1913	0.045*	
C35	0.4212 (5)	0.1555 (4)	0.2612 (2)	0.0477 (13)	
H35A	0.4851	0.1311	0.2372	0.057*	
H35B	0.4124	0.2186	0.2519	0.057*	
C36	0.4591 (5)	0.1375 (3)	0.3380 (2)	0.0444 (12)	
H36A	0.4615	0.1919	0.3640	0.053*	
H36B	0.5439	0.1090	0.3496	0.053*	
C37	0.1943 (5)	−0.0362 (4)	0.2786 (3)	0.0520 (14)	
H37A	0.1649	−0.0544	0.2318	0.078*	
H37B	0.1264	−0.0033	0.2933	0.078*	
H37C	0.2156	−0.0875	0.3072	0.078*	
C38	0.4259 (5)	−0.0355 (4)	0.2702 (3)	0.0526 (14)	
H38A	0.4493	−0.0782	0.3063	0.079*	
H38B	0.5002	0.0011	0.2682	0.079*	
H38C	0.3984	−0.0656	0.2272	0.079*	
C39	0.3849 (5)	0.0237 (3)	0.4182 (2)	0.0449 (12)	
H39A	0.3237	−0.0236	0.4183	0.067*	
H39B	0.3830	0.0626	0.4561	0.067*	
H39C	0.4712	−0.0004	0.4224	0.067*	

### Atomic displacement parameters (Å^2^)

**Table:**

	*U*^11^	*U*^22^	*U*^33^	*U*^12^	*U*^13^	*U*^23^
Ir1	0.03940 (11)	0.03886 (12)	0.03298 (10)	0.00425 (9)	0.01573 (7)	0.00030 (9)
O1	0.0318 (18)	0.049 (2)	0.0268 (17)	0.0043 (14)	0.0096 (14)	−0.0017 (14)
O2	0.0458 (18)	0.043 (2)	0.0357 (17)	0.0114 (15)	0.0133 (14)	0.0069 (15)
N1	0.042 (2)	0.040 (2)	0.045 (3)	0.0126 (19)	0.017 (2)	0.0055 (19)
N2	0.038 (2)	0.039 (2)	0.031 (2)	−0.0003 (18)	0.0096 (17)	0.0057 (17)
C1	0.049 (3)	0.056 (4)	0.041 (3)	0.004 (3)	0.013 (3)	−0.005 (3)
C2	0.046 (3)	0.054 (4)	0.064 (4)	0.003 (3)	0.012 (3)	−0.011 (3)
C3	0.042 (3)	0.049 (4)	0.081 (5)	0.001 (3)	0.013 (3)	0.002 (3)
C4	0.050 (3)	0.052 (4)	0.064 (4)	0.002 (3)	0.022 (3)	0.013 (3)
C5	0.037 (3)	0.043 (3)	0.052 (3)	0.007 (2)	0.018 (2)	0.008 (2)
C6	0.038 (3)	0.044 (3)	0.042 (3)	0.009 (2)	0.019 (2)	0.006 (2)
C7	0.052 (3)	0.061 (4)	0.047 (3)	0.001 (3)	0.024 (3)	0.009 (3)
C8	0.058 (3)	0.075 (4)	0.046 (3)	0.016 (3)	0.026 (3)	0.012 (3)
C9	0.063 (4)	0.063 (4)	0.044 (3)	0.016 (3)	0.022 (3)	−0.006 (3)
C10	0.052 (3)	0.057 (4)	0.046 (3)	0.006 (3)	0.023 (3)	−0.001 (3)
C11	0.044 (3)	0.045 (3)	0.043 (3)	0.010 (2)	0.020 (2)	0.002 (2)
C12	0.051 (3)	0.060 (4)	0.042 (3)	−0.002 (3)	0.015 (3)	0.000 (3)
C13	0.056 (4)	0.077 (5)	0.055 (4)	−0.012 (3)	0.007 (3)	0.007 (3)
C14	0.110 (5)	0.053 (4)	0.044 (3)	−0.027 (4)	0.011 (4)	−0.001 (3)
C15	0.073 (4)	0.043 (3)	0.049 (3)	0.005 (3)	0.017 (3)	−0.005 (3)
C16	0.069 (4)	0.047 (3)	0.027 (3)	0.000 (3)	0.018 (2)	0.006 (2)
C17	0.059 (3)	0.045 (3)	0.036 (3)	0.015 (3)	0.020 (3)	0.001 (2)
C18	0.088 (5)	0.049 (4)	0.052 (4)	0.019 (3)	0.020 (3)	0.000 (3)
C19	0.059 (4)	0.088 (6)	0.062 (4)	0.027 (4)	0.015 (3)	0.009 (4)
C20	0.051 (4)	0.066 (5)	0.091 (5)	0.018 (3)	0.017 (3)	0.004 (4)
C21	0.049 (3)	0.058 (4)	0.073 (4)	0.010 (3)	0.017 (3)	0.005 (3)
C22	0.045 (3)	0.042 (3)	0.036 (3)	0.010 (2)	0.018 (2)	−0.002 (2)
C23	0.035 (2)	0.037 (3)	0.029 (2)	0.002 (2)	0.012 (2)	0.002 (2)
C24	0.042 (3)	0.034 (3)	0.032 (2)	0.002 (2)	0.004 (2)	0.001 (2)
C25	0.051 (3)	0.041 (3)	0.033 (2)	−0.003 (2)	0.011 (2)	0.006 (2)
C26	0.076 (4)	0.055 (4)	0.039 (3)	−0.003 (3)	0.017 (3)	0.011 (3)
C27	0.078 (4)	0.080 (5)	0.027 (3)	−0.001 (3)	0.000 (3)	0.013 (3)
C28	0.056 (3)	0.084 (5)	0.046 (3)	0.012 (3)	−0.008 (3)	0.002 (3)
C29	0.044 (3)	0.069 (4)	0.044 (3)	0.005 (3)	0.008 (2)	0.006 (3)
C30	0.038 (3)	0.039 (3)	0.024 (2)	0.000 (2)	0.008 (2)	0.002 (2)
C31	0.033 (2)	0.035 (3)	0.027 (2)	−0.0026 (19)	0.0097 (19)	−0.001 (2)
C32	0.037 (3)	0.040 (3)	0.029 (2)	0.002 (2)	0.009 (2)	0.002 (2)
C33	0.041 (3)	0.041 (3)	0.032 (3)	0.003 (2)	0.009 (2)	0.001 (2)
C34	0.045 (3)	0.044 (3)	0.025 (2)	0.007 (2)	0.009 (2)	0.001 (2)
C35	0.051 (3)	0.054 (3)	0.044 (3)	0.000 (2)	0.025 (3)	0.007 (2)
C36	0.039 (3)	0.054 (3)	0.040 (3)	−0.004 (2)	0.007 (2)	−0.002 (2)
C37	0.055 (3)	0.051 (3)	0.049 (3)	−0.011 (3)	0.007 (3)	0.002 (3)
C38	0.064 (3)	0.051 (3)	0.042 (3)	0.020 (3)	0.009 (3)	−0.002 (3)
C39	0.041 (3)	0.055 (3)	0.038 (3)	0.004 (2)	0.005 (2)	0.008 (2)

### Geometric parameters (Å, °)

**Table:**

Ir1—O1	2.171 (3)	C19—H19	0.9300
Ir1—O2	2.153 (3)	C19—C20	1.358 (9)
Ir1—N1	2.033 (4)	C20—H20	0.9300
Ir1—N2	2.032 (4)	C20—C21	1.358 (7)
Ir1—C11	2.001 (5)	C21—H21	0.9300
Ir1—C22	1.996 (5)	C21—C22	1.402 (6)
O1—C31	1.272 (5)	C23—C24	1.512 (6)
O2—C23	1.285 (5)	C23—C30	1.382 (6)
N1—C1	1.349 (6)	C24—C25	1.390 (6)
N1—C5	1.392 (6)	C24—C29	1.384 (7)
N2—C12	1.349 (6)	C25—H25	0.9300
N2—C16	1.358 (6)	C25—C26	1.388 (7)
C1—H1	0.9300	C26—H26	0.9300
C1—C2	1.383 (7)	C26—C27	1.334 (8)
C2—H2	0.9300	C27—H27	0.9300
C2—C3	1.404 (7)	C27—C28	1.376 (8)
C3—H3	0.9300	C28—H28	0.9300
C3—C4	1.368 (8)	C28—C29	1.377 (7)
C4—H4	0.9300	C29—H29	0.9300
C4—C5	1.377 (7)	C30—C31	1.406 (6)
C5—C6	1.459 (7)	C30—C34	1.526 (6)
C6—C7	1.396 (6)	C31—C32	1.522 (6)
C6—C11	1.401 (6)	C32—C33	1.549 (6)
C7—H7	0.9300	C32—C36	1.564 (6)
C7—C8	1.388 (8)	C32—C39	1.500 (6)
C8—H8	0.9300	C33—C34	1.553 (6)
C8—C9	1.364 (8)	C33—C37	1.524 (6)
C9—H9	0.9300	C33—C38	1.518 (6)
C9—C10	1.380 (7)	C34—H34	0.9800
C10—H10	0.9300	C34—C35	1.534 (6)
C10—C11	1.384 (7)	C35—H35A	0.9700
C12—H12	0.9300	C35—H35B	0.9700
C12—C13	1.383 (8)	C35—C36	1.523 (6)
C13—H13	0.9300	C36—H36A	0.9700
C13—C14	1.371 (8)	C36—H36B	0.9700
C14—H14	0.9300	C37—H37A	0.9600
C14—C15	1.377 (8)	C37—H37B	0.9600
C15—H15	0.9300	C37—H37C	0.9600
C15—C16	1.356 (7)	C38—H38A	0.9600
C16—C17	1.487 (7)	C38—H38B	0.9600
C17—C18	1.404 (7)	C38—H38C	0.9600
C17—C22	1.411 (7)	C39—H39A	0.9600
C18—H18	0.9300	C39—H39B	0.9600
C18—C19	1.410 (8)	C39—H39C	0.9600
			
?···?	?		
			
O2—Ir1—O1	88.99 (11)	C21—C20—H20	119.5
N1—Ir1—O1	87.18 (13)	C20—C21—H21	118.5
N1—Ir1—O2	93.82 (14)	C20—C21—C22	122.9 (6)
N2—Ir1—O1	94.93 (14)	C22—C21—H21	118.5
N2—Ir1—O2	91.04 (13)	C17—C22—Ir1	114.5 (4)
N2—Ir1—N1	174.74 (14)	C21—C22—Ir1	129.6 (4)
C11—Ir1—O1	90.47 (15)	C21—C22—C17	115.9 (5)
C11—Ir1—O2	174.84 (17)	O2—C23—C24	113.5 (4)
C11—Ir1—N1	81.03 (19)	O2—C23—C30	125.1 (4)
C11—Ir1—N2	94.12 (18)	C30—C23—C24	121.3 (4)
C22—Ir1—O1	175.56 (16)	C25—C24—C23	122.2 (4)
C22—Ir1—O2	90.97 (15)	C29—C24—C23	119.8 (4)
C22—Ir1—N1	97.25 (18)	C29—C24—C25	117.9 (4)
C22—Ir1—N2	80.63 (19)	C24—C25—H25	120.1
C22—Ir1—C11	89.96 (19)	C26—C25—C24	119.8 (5)
C31—O1—Ir1	120.9 (3)	C26—C25—H25	120.1
C23—O2—Ir1	126.1 (3)	C25—C26—H26	119.4
C1—N1—Ir1	125.7 (3)	C27—C26—C25	121.2 (5)
C1—N1—C5	119.1 (4)	C27—C26—H26	119.4
C5—N1—Ir1	115.2 (3)	C26—C27—H27	119.9
C12—N2—Ir1	124.4 (3)	C26—C27—C28	120.2 (5)
C12—N2—C16	118.5 (5)	C28—C27—H27	119.9
C16—N2—Ir1	116.8 (3)	C27—C28—H28	120.2
N1—C1—H1	118.3	C27—C28—C29	119.5 (5)
N1—C1—C2	123.5 (5)	C29—C28—H28	120.2
C2—C1—H1	118.3	C24—C29—H29	119.4
C1—C2—H2	121.3	C28—C29—C24	121.2 (5)
C1—C2—C3	117.5 (5)	C28—C29—H29	119.4
C3—C2—H2	121.3	C23—C30—C31	127.7 (4)
C2—C3—H3	120.5	C23—C30—C34	128.4 (4)
C4—C3—C2	118.9 (5)	C31—C30—C34	104.0 (4)
C4—C3—H3	120.5	O1—C31—C30	131.0 (4)
C3—C4—H4	118.7	O1—C31—C32	121.1 (4)
C3—C4—C5	122.6 (5)	C30—C31—C32	107.8 (4)
C5—C4—H4	118.7	C31—C32—C33	100.3 (4)
N1—C5—C6	113.4 (4)	C31—C32—C36	103.6 (4)
C4—C5—N1	118.4 (5)	C33—C32—C36	101.3 (3)
C4—C5—C6	128.2 (5)	C39—C32—C31	116.6 (4)
C7—C6—C5	122.9 (5)	C39—C32—C33	118.5 (4)
C7—C6—C11	121.3 (5)	C39—C32—C36	114.1 (4)
C11—C6—C5	115.8 (4)	C32—C33—C34	92.5 (4)
C6—C7—H7	120.1	C37—C33—C32	113.4 (3)
C8—C7—C6	119.8 (5)	C37—C33—C34	113.2 (4)
C8—C7—H7	120.1	C38—C33—C32	113.9 (4)
C7—C8—H8	120.3	C38—C33—C34	115.3 (4)
C9—C8—C7	119.5 (5)	C38—C33—C37	108.0 (4)
C9—C8—H8	120.3	C30—C34—C33	102.4 (3)
C8—C9—H9	119.9	C30—C34—H34	114.7
C8—C9—C10	120.3 (6)	C30—C34—C35	107.4 (4)
C10—C9—H9	119.9	C33—C34—H34	114.7
C9—C10—H10	118.7	C35—C34—C33	101.4 (4)
C9—C10—C11	122.7 (5)	C35—C34—H34	114.7
C11—C10—H10	118.7	C34—C35—H35A	111.2
C6—C11—Ir1	114.5 (4)	C34—C35—H35B	111.2
C10—C11—Ir1	129.0 (4)	H35A—C35—H35B	109.1
C10—C11—C6	116.4 (4)	C36—C35—C34	102.7 (4)
N2—C12—H12	118.9	C36—C35—H35A	111.2
N2—C12—C13	122.1 (5)	C36—C35—H35B	111.2
C13—C12—H12	118.9	C32—C36—H36A	111.0
C12—C13—H13	120.6	C32—C36—H36B	111.0
C14—C13—C12	118.8 (6)	C35—C36—C32	104.0 (4)
C14—C13—H13	120.6	C35—C36—H36A	111.0
C13—C14—H14	120.6	C35—C36—H36B	111.0
C13—C14—C15	118.7 (6)	H36A—C36—H36B	109.0
C15—C14—H14	120.6	C33—C37—H37A	109.5
C14—C15—H15	119.5	C33—C37—H37B	109.5
C16—C15—C14	120.9 (6)	C33—C37—H37C	109.5
C16—C15—H15	119.5	H37A—C37—H37B	109.5
N2—C16—C17	112.5 (5)	H37A—C37—H37C	109.5
C15—C16—N2	120.9 (5)	H37B—C37—H37C	109.5
C15—C16—C17	126.6 (5)	C33—C38—H38A	109.5
C18—C17—C16	123.1 (5)	C33—C38—H38B	109.5
C18—C17—C22	121.8 (5)	C33—C38—H38C	109.5
C22—C17—C16	115.1 (4)	H38A—C38—H38B	109.5
C17—C18—H18	120.8	H38A—C38—H38C	109.5
C17—C18—C19	118.4 (6)	H38B—C38—H38C	109.5
C19—C18—H18	120.8	C32—C39—H39A	109.5
C18—C19—H19	120.0	C32—C39—H39B	109.5
C20—C19—C18	120.0 (6)	C32—C39—H39C	109.5
C20—C19—H19	120.0	H39A—C39—H39B	109.5
C19—C20—H20	119.5	H39A—C39—H39C	109.5
C21—C20—C19	121.0 (6)	H39B—C39—H39C	109.5
			
Ir1—O1—C31—C30	4.1 (7)	C11—Ir1—N1—C5	−1.2 (3)
Ir1—O1—C31—C32	−178.1 (3)	C11—Ir1—N2—C12	90.3 (4)
Ir1—O2—C23—C24	179.6 (3)	C11—Ir1—N2—C16	−83.1 (3)
Ir1—O2—C23—C30	3.6 (7)	C11—Ir1—C22—C17	90.6 (4)
Ir1—N1—C1—C2	−179.7 (4)	C11—Ir1—C22—C21	−89.3 (5)
Ir1—N1—C5—C4	−179.7 (4)	C11—C6—C7—C8	0.5 (7)
Ir1—N1—C5—C6	−0.8 (5)	C12—N2—C16—C15	−1.5 (7)
Ir1—N2—C12—C13	−172.6 (4)	C12—N2—C16—C17	179.0 (4)
Ir1—N2—C16—C15	172.3 (4)	C12—C13—C14—C15	−1.5 (8)
Ir1—N2—C16—C17	−7.2 (5)	C13—C14—C15—C16	0.7 (8)
O1—Ir1—O2—C23	−0.2 (4)	C14—C15—C16—N2	0.9 (8)
O1—Ir1—N1—C1	−90.4 (4)	C14—C15—C16—C17	−179.7 (5)
O1—Ir1—N1—C5	89.7 (3)	C15—C16—C17—C18	6.2 (8)
O1—Ir1—N2—C12	−0.5 (4)	C15—C16—C17—C22	−175.4 (5)
O1—Ir1—N2—C16	−173.9 (3)	C16—N2—C12—C13	0.6 (7)
O1—Ir1—C11—C6	−84.1 (4)	C16—C17—C18—C19	−179.9 (5)
O1—Ir1—C11—C10	92.7 (5)	C16—C17—C22—Ir1	0.9 (5)
O1—Ir1—C22—C17	−5(2)	C16—C17—C22—C21	−179.2 (4)
O1—Ir1—C22—C21	175.2 (16)	C17—C18—C19—C20	−1.2 (9)
O1—C31—C32—C33	146.6 (4)	C18—C17—C22—Ir1	179.4 (4)
O1—C31—C32—C36	−109.0 (4)	C18—C17—C22—C21	−0.8 (7)
O1—C31—C32—C39	17.2 (6)	C18—C19—C20—C21	−0.2 (10)
O2—Ir1—O1—C31	−3.3 (3)	C19—C20—C21—C22	1.2 (9)
O2—Ir1—N1—C1	−1.6 (4)	C20—C21—C22—Ir1	179.2 (5)
O2—Ir1—N1—C5	178.5 (3)	C20—C21—C22—C17	−0.7 (8)
O2—Ir1—N2—C12	−89.6 (4)	C22—Ir1—O1—C31	−92.8 (19)
O2—Ir1—N2—C16	97.0 (3)	C22—Ir1—O2—C23	175.3 (4)
O2—Ir1—C11—C6	−0.1 (19)	C22—Ir1—N1—C1	89.9 (4)
O2—Ir1—C11—C10	176.7 (14)	C22—Ir1—N1—C5	−90.0 (3)
O2—Ir1—C22—C17	−94.5 (4)	C22—Ir1—N2—C12	179.6 (4)
O2—Ir1—C22—C21	85.7 (4)	C22—Ir1—N2—C16	6.2 (3)
O2—C23—C24—C25	143.4 (4)	C22—Ir1—C11—C6	100.3 (4)
O2—C23—C24—C29	−31.9 (6)	C22—Ir1—C11—C10	−82.8 (5)
O2—C23—C30—C31	−4.2 (8)	C22—C17—C18—C19	1.7 (8)
O2—C23—C30—C34	176.9 (4)	C23—C24—C25—C26	−178.2 (4)
N1—Ir1—O1—C31	90.6 (3)	C23—C24—C29—C28	177.3 (5)
N1—Ir1—O2—C23	−87.3 (4)	C23—C30—C31—O1	−0.3 (8)
N1—Ir1—N2—C12	112.9 (16)	C23—C30—C31—C32	−178.2 (5)
N1—Ir1—N2—C16	−60.5 (17)	C23—C30—C34—C33	−146.9 (5)
N1—Ir1—C11—C6	3.0 (3)	C23—C30—C34—C35	106.7 (5)
N1—Ir1—C11—C10	179.8 (5)	C24—C23—C30—C31	−179.9 (4)
N1—Ir1—C22—C17	171.5 (3)	C24—C23—C30—C34	1.2 (8)
N1—Ir1—C22—C21	−8.3 (5)	C24—C25—C26—C27	0.6 (8)
N1—C1—C2—C3	−1.6 (8)	C25—C24—C29—C28	1.7 (8)
N1—C5—C6—C7	−177.8 (4)	C25—C26—C27—C28	2.7 (9)
N1—C5—C6—C11	3.3 (6)	C26—C27—C28—C29	−3.7 (9)
N2—Ir1—O1—C31	−94.2 (3)	C27—C28—C29—C24	1.5 (9)
N2—Ir1—O2—C23	94.7 (4)	C29—C24—C25—C26	−2.8 (7)
N2—Ir1—N1—C1	155.9 (15)	C30—C23—C24—C25	−40.4 (7)
N2—Ir1—N1—C5	−24.0 (18)	C30—C23—C24—C29	144.2 (5)
N2—Ir1—C11—C6	−179.0 (3)	C30—C31—C32—C33	−35.2 (4)
N2—Ir1—C11—C10	−2.2 (5)	C30—C31—C32—C36	69.2 (4)
N2—Ir1—C22—C17	−3.6 (3)	C30—C31—C32—C39	−164.6 (4)
N2—Ir1—C22—C21	176.6 (5)	C30—C34—C35—C36	67.0 (5)
N2—C12—C13—C14	0.9 (8)	C31—C30—C34—C33	34.0 (5)
N2—C16—C17—C18	−174.3 (4)	C31—C30—C34—C35	−72.4 (4)
N2—C16—C17—C22	4.1 (6)	C31—C32—C33—C34	51.7 (4)
C1—N1—C5—C4	0.4 (7)	C31—C32—C33—C37	−65.0 (5)
C1—N1—C5—C6	179.3 (4)	C31—C32—C33—C38	170.9 (4)
C1—C2—C3—C4	2.4 (8)	C31—C32—C36—C35	−71.1 (4)
C2—C3—C4—C5	−1.9 (8)	C32—C33—C34—C30	−52.6 (4)
C3—C4—C5—N1	0.5 (8)	C32—C33—C34—C35	58.3 (4)
C3—C4—C5—C6	−178.2 (5)	C33—C32—C36—C35	32.6 (5)
C4—C5—C6—C7	1.0 (8)	C33—C34—C35—C36	−40.0 (4)
C4—C5—C6—C11	−177.8 (5)	C34—C30—C31—O1	178.8 (5)
C5—N1—C1—C2	0.2 (7)	C34—C30—C31—C32	0.8 (5)
C5—C6—C7—C8	−178.4 (4)	C34—C35—C36—C32	4.4 (5)
C5—C6—C11—Ir1	−4.3 (5)	C36—C32—C33—C34	−54.5 (4)
C5—C6—C11—C10	178.4 (4)	C36—C32—C33—C37	−171.2 (4)
C6—C7—C8—C9	−0.6 (8)	C36—C32—C33—C38	64.7 (5)
C7—C6—C11—Ir1	176.8 (4)	C37—C33—C34—C30	64.3 (5)
C7—C6—C11—C10	−0.5 (7)	C37—C33—C34—C35	175.2 (4)
C7—C8—C9—C10	0.8 (8)	C38—C33—C34—C30	−170.6 (4)
C8—C9—C10—C11	−0.9 (8)	C38—C33—C34—C35	−59.7 (5)
C9—C10—C11—Ir1	−176.1 (4)	C39—C32—C33—C34	179.8 (4)
C9—C10—C11—C6	0.7 (7)	C39—C32—C33—C37	63.1 (5)
C11—Ir1—O1—C31	171.6 (3)	C39—C32—C33—C38	−61.0 (5)
C11—Ir1—O2—C23	−84.2 (17)	C39—C32—C36—C35	161.1 (4)
C11—Ir1—N1—C1	178.7 (4)		
